# Accidental Synthetic Cannabinoid Poisoning in a Pediatric Patient: A Case Report

**DOI:** 10.7759/cureus.74936

**Published:** 2024-12-01

**Authors:** Andrew J Gonedes, Eric Boccio

**Affiliations:** 1 Emergency Medicine, Memorial Healthcare System, Hollywood, USA

**Keywords:** cannabinoid ingestion, cannabinoid receptors, cannabis edible, pediatric ingestion, tetrahydrocannabinol effects, tetrahydrocannabinol (thc)

## Abstract

A six-year-old boy presented to the pediatric emergency department following an accidental ingestion of a synthetic cannabinoid. The child ingested an edible product containing tetrahydrocannabinol (THC) and presented with lethargy, altered mental status, and increased muscle tone. The airway was protected, and the patient was breathing spontaneously. Initial assessment included a thorough history provided by the caregivers, which confirmed ingestion of cannabinoid gummies approximately two hours prior to presentation. An electrocardiogram demonstrated sinus tachycardia with a prolonged QTc. Toxicology screening was presumed positive for cannabinoids. Therapeutic management focused on supportive care. The patient was admitted to the hospital and discharged on hospital day two with no neurologic sequelae. The caregivers were provided with expectant management and counseled regarding safe storage and use of cannabis and cannabis-derived products. This case report discusses the growing incidence of cannabis exposure in the pediatric population while highlighting the biochemistry, clinical presentation, and therapeutic management of accidental ingestion of synthetic cannabinoids.

## Introduction

Cannabinoids refer to the group of chemical compounds that interact with the human body's endocannabinoid system. They include phytocannabinoids, which are found naturally in the cannabis plant; endocannabinoids, which are produced naturally by the body; and synthetic cannabinoids, which are artificially produced and designed to mimic the effects of natural cannabinoids. The main cannabinoids include tetrahydrocannabinol (THC) and cannabidiol (CBD), which are typically consumed for recreational purposes or self-treatment of conditions such as chronic pain, anxiety, and insomnia. To date, the United States Food and Drug Administration has not approved the cannabis plant for medical use; however, there are several drugs that contain cannabis-derived cannabinoids and are used to treat seizures associated with Lennox-Gastaut syndrome or Dravet syndrome (Epidiolex), nausea secondary to chemotherapy (Marinol), and anorexia associated with acquired immunodeficiency syndrome (Syndros).

Cannabinoids can pose risks to pediatric patients, particularly when consumed in excessive amounts. Accidental ingestion of cannabis products has increased among children, most likely as a result of the increased ease of access following widespread state legalization of marijuana for recreational use [1]. A retrospective analysis of National Poison Data System reports filed between 2017 and 2021 revealed 7,043 exposures to edible cannabis products in children less than six years of age, with almost all events occurring in a residential setting [2]. Seventy percent of cases involved central nervous system depression, while 22.7% of patients were admitted to the hospital [2]. This report described a 1375% increase in the incidence of pediatric accidental cannabis ingestion over the five-year period, a trend that is expected to continue to increase at an accelerated rate [2].

While the risk of a fatal overdose from cannabis is relatively low compared to substances like opioids, there are still concerns regarding their overall short- and long-term impact on children. Younger children are particularly susceptible to cannabis toxicity due to their lower total body weight [3]. Symptoms of cannabinoid toxicity can include drowsiness, confusion, altered mental status, and poor motor coordination, as well as nausea and vomiting, agitation, anxiety, paranoia, tachycardia, and blood pressure lability [4]. Clonus and muscle rigidity have been described in limited studies and appear to be associated with new synthetic cannabinoids, suggesting that their side effect profile is wide and unpredictable.

Cannabinoids may influence various neurotransmitter systems, including dopamine, serotonin, and glutamate, leading to a wide range of psychological and physiological effects involving mood, cognition, and pain perception. High doses may increase the risk of psychosis or exacerbate underlying mental health issues. Studies indicate that children and adolescents exposed to cannabinoids are at a higher risk for developing mental health issues, including anxiety, depression, and psychosis [5-6]. We present the case of a synthetic cannabinoid overdose in a pediatric patient presenting to the emergency department.

## Case presentation

A six-year-old Hispanic boy (weight: 25 kilograms, height: 120 centimeters) with no significant past medical history presented to the pediatric emergency department with altered mental status and muscle pain after a suspected cannabinoid ingestion. Per parents, the patient attended a family birthday party earlier in the evening. Upon returning home, the patient went to sleep and awoke complaining of extreme pain throughout all four extremities. The patient was last seen normal two hours prior to presentation. The parents noted that the patient had increased muscle tone in the bilateral upper and lower extremities and experienced multiple episodes of non-bloody and non-bilious emesis. The family had a high suspicion of accidental cannabinoid gummy ingestion but were uncertain of the THC/CBD concentration, the total amount consumed, and the time of ingestion.

**Figure 1:**
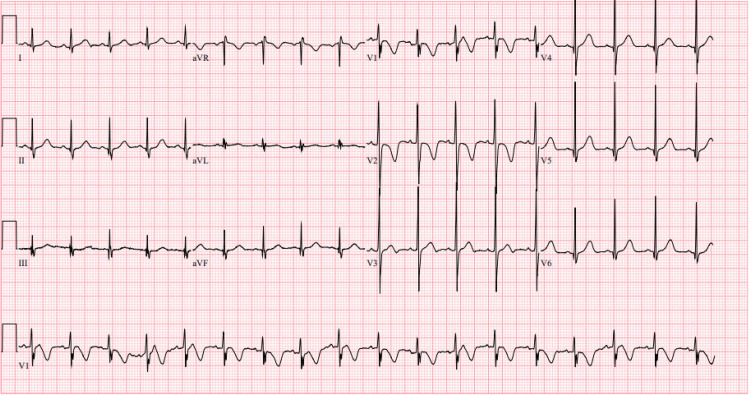
Initial electrocardiogram obtained upon arrival to the pediatric emergency department The electrocardiogram was interpreted as an age-specific normal sinus rhythm (ventricular rate 107 beats per minute) with a normal PR interval (118 milliseconds) and prolonged QTc (451 milliseconds).

Initial vital signs revealed blood pressure of 103/67 millimeters of mercury, heart rate of 91 beats per minute, respiratory rate of 20 breaths per minute, peripheral oxygen saturation of 100% on room air, and temperature of 36.8 degrees Celsius, oral. An electrocardiogram demonstrated a normal sinus rhythm with a ventricular rate of 107 beats per minute and a QTc of 486 milliseconds (Figure 1). Basic labs, including a complete blood count, comprehensive metabolic panel, and prothrombin time test/international normalized ratio, were within normal limits. Troponin I and creatine kinase levels were also within normal limits. Ethanol, acetaminophen, and salicylate levels were undetectable while a urine toxicology screen was presumed positive for cannabinoids.

The patient was administered 0.05 milligrams/kilogram of lorazepam intravenously once for agitation and increased muscle tone. The local Poison Control Center was contacted, and the case was presented. Given concerns for a possible anticholinergic co-ingestion, it was recommended that the patient be observed for a 24-hour period. The family denied any recent cold and cough medication ingestion. On reevaluation, the patient was noted to be rigid throughout. He was unable to extend any extremities and was minimally responsive with dilated and sluggishly reactive pupils. The patient was administered a second dose of 0.05 milligrams/kilogram lorazepam intravenously. The patient's mental status and physical examination returned to baseline, and the patient did not have any recurrence of symptoms while in the emergency department.

While inpatient, arterial blood gas demonstrated a mild hypercapnia. The patient was started on noninvasive pressure control via a facemask. Clinical reassessment and repeat blood gas demonstrated an improvement in respiratory status; noninvasive ventilation was discontinued, and the patient was transitioned to room air. The patient remained on room air and maintained oxygen saturation above 92% while exhibiting no increased work of breathing. Repeat electrocardiogram demonstrated improvement in the QTc prolongation. Cardiology was consulted due to the concern of high-voltage QRS complexes, and an echocardiogram was recommended. The echocardiogram was unremarkable with no recommendations for future follow-up needed. The patient underwent close neurologic monitoring; as ingestion effects continued to wear off, the patient returned to his neurologic baseline. He remained at a neurologic baseline with no sequelae.

On hospital day two, the patient remained hemodynamically stable, afebrile, and on room air, maintaining goal saturations with a comfortable-appearing work of breathing. The patient tolerated oral intake without emesis or diarrhea. His neurologic status remained at baseline per family with no sequelae. He was medically cleared and discharged home with return precautions. The family was counseled regarding safe storage and use of all recreational drugs in the home.

## Discussion

Cannabinoids primarily exert their effects by interacting with the endocannabinoid system in the body, which consists of cannabinoid receptors, endogenous cannabinoids (endocannabinoids), and enzymes that synthesize and degrade these compounds [7]. The two main types of cannabinoid receptors are cannabinoid 1 (CB1) and cannabinoid 2 (CB2). Cannabinoid 1 receptors are mainly found in the brain and central nervous system and are responsible for the psychoactive effects of THC. Activation of CB1 receptors can influence mood, memory, appetite, and pain sensation [8]. Cannabinoid 2 receptors are primarily located in the peripheral nervous system and immune cells. They are involved in regulating immune response and inflammation. Cannabinoids that activate CB2 receptors may have therapeutic effects without the psychoactive effects associated with CB1 activation [8].

Delta-8-tetrahydrocannabinol (Delta-8-THC) is a cannabinoid similar to Delta-9-tetrahydrocannabinol (Delta-9-THC), the primary psychoactive compound in cannabis. Its legality and safety profile are still under scrutiny [9]. It is often marketed as providing a mild euphoric effect, with potentially fewer side effects [9]. Another isomer of THC, Delta-10-tetrahydrocannabinol (Delta-10-THC), is said to be less potent than Delta-9-THC. Delta-10-THC is becoming more popular in various cannabis products, though its effects and safety are still being studied [10]. Tetrahydrocannabiphorol (THCP) is a recently discovered cannabinoid that may have a significantly higher potency than Delta-9-THC. Research is ongoing to understand its effects and therapeutic potential [11].

Three cannabinoid variants have been reported. Cannabidivarin (CBDV) is similar to CBD but with a different chemical structure. It is being studied for its potential therapeutic benefits, particularly for neurological conditions [12-14]. Cannabigerol (CBG), often referred to as the "mother" cannabinoid because it's a precursor to other cannabinoids, is gaining interest for its potential anti-inflammatory and neuroprotective properties [15]. Cannabichromene (CBC) is another non-psychoactive cannabinoid with potential therapeutic benefits. It is being explored for its effects on pain and inflammation [16]. Cannabis breeders are continually developing new hybrid strains that combine various cannabinoids and terpenes, the aromatic compounds in cannabis, to produce unique effects and flavors for specific medical uses [17].

Cannabinoid overdose in pediatric patients is relatively uncommon but has been increasingly reported, particularly with the rise in the availability of cannabis products [18]. There has been a noticeable increase in cases of cannabinoid exposure in children, especially due to the availability of cannabis edibles and other products that are attractive to young children. In recent years, the number of emergency department visits for cannabis-related issues has grown, reflecting this trend. The majority of cannabinoid-related incidents in children involve accidental ingestion of products like edibles. These products, often designed to be appealing and flavorful, can inadvertently be consumed by children [18].

Mild symptoms of cannabis toxicity normally include drowsiness, lethargy, mild confusion, and changes in behavior. Gastrointestinal issues such as nausea and vomiting can also occur, as well as tachycardia and labile blood pressure [19]. In rare cases, such as the case presented, high doses of unknown synthetic cannabinoids can cause severe muscle rigidity and posturing with a mixed picture of overdose symptoms; these symptoms typically respond to benzodiazepines [20]. In more severe cases, symptoms may escalate to include intense agitation, paranoia, hallucinations, severe drowsiness, and difficulty with motor coordination [19].

The treatment for cannabis toxicity generally focuses on supportive care and symptom management. Most cannabis toxicity cases are self-resolving. Inhaled THC reaches a maximum plasma concentration within minutes, and psychotropic effects peak within 15-30 minutes and taper after two to three hours [21]. When ingested orally, psychotropic effects present after 60-90 minutes with a maximum effect after two to three hours and taper after four to 12 hours, depending on concentration and total volume ingested [21]. Patients should be evaluated for possible co-ingestions. Tachycardia and labile blood pressure should be monitored closely. An electrocardiogram should be obtained to assess for potential cardiac dysrhythmias. Anxiety, agitation, and paranoia are common, so maintaining a comfortable setting and administering anxiolytics as necessary may be beneficial. Nausea and vomiting can be treated with antiemetics, and, if the patient can successfully tolerate oral intake, fluids per os can be given to alleviate dry mouth. In certain instances, activated charcoal may be given within one hour of ingestion to reduce absorption. If the patient demonstrates signs of psychosis, extreme agitation, or distress, sedation may be indicated [22]. Admission and inpatient observation may be necessary if there is a high clinical suspicion for co-ingestion.

## Conclusions

Synthetic cannabinoid poisoning secondary to accidental ingestion of edibles in pediatric patients has become increasingly prevalent due to the rising availability of edible cannabis and cannabis-derived products. These products are often marketed to resemble popular food and candies or sold in brightly colored packaging that could appeal to children. Reports indicate that pediatric emergency department visits related to cannabinoid ingestion among children have significantly increased, prompting concerns about their safety. Higher concentrations of THC increase the risk of more pronounced side effects, and children are particularly vulnerable due to their smaller body size and lower tolerance compared to adults. Most cases of cannabinoid toxicity are managed with supportive care, and symptoms are self-limiting. Muscle rigidity is more commonly associated with synthetic cannabinoids and may lead to complications such as respiratory distress or injury due to falls.
